# Supplementary material for: A mixed methods analysis of youth mental health intervention feasibility and acceptability in a North American city: Perspectives from Seattle, Washington
Source: PLoS One. 2024 Mar 14;19(3):e0288214. doi: 10.1371/journal.pone.0288214 (PMC10939237; doi:10.1371/journal.pone.0288214)
Supplement: S1 Appendix — (DOCX) [file pone.0288214.s001.docx]

**S1 Appendix. Quantitative Survey Variables Data Dictionary**

| **Name** | **Description** | **Measurement Unit** | **Data Type** |
| --- | --- | --- | --- |
| **Socio-Demographic Variables** | | | |
| Age Category | The age category of the participants at the time of the study | Categorical (Years) | Categories:   - 17-19 - 20-24 - 25-34 - 45-54 - 55-64 |
| Gender Identity | The gender identity of the participants | Categorical | Categories:   - Woman: Participants who identify as female. - Man: Participants who identify as male - Nonbinary: Participants who identify outside of the traditional gender binary |
| Race/Ethnicity | The racial and ethnic group or groups of the participants | Categorical | Categories:   - Alaska Native - American Indian/Native American - Asian - Asian – South Asian - Asian – Southeast Asian - Black/African American - Black African - Hispanic/Latinx/Chicanx - Middle Eastern - Native Hawaiian - Pacific Islander - White/European American - A racial/ethnic identity not listed |
| Youth Identification | The identification of participants as youth or non-youth | Binary | Categories   - Youth: Participants who identify themselves as belonging to the youth category - Non-Youth: Participants who do not identify themselves as belonging to the youth category |
| Employment Status | The employment status of the participants | Categorical | Categories:   - Studying - Employed - Self-employed/freelance - Interning - Part-time - Unemployed-looking for work - Unemployed-not looking for work - Homemaker - Military - Retired - Not able to work - Other |
| Education Level | The educational attainment status of the participants | Categorical | Categories:   - No formal education - Pre-school to 8th grade - Some high school, no diploma - High school graduate, diploma, or equivalent (e.g., GED) - Some college credit, no degree - Trade/technical/vocational training - Associate degree - Bachelor’s degree - Master’s degree - Professional degree - Doctorate degree |
| Residence Status (i.e., living in South Seattle) | Residence in South Seattle or other regions of the city | Binary | Categories:   - Participant identifies as living in South Seattle - Participant does not identify as living in South Seattle |
| Zip code | The zip code of the participants' residential address | Discrete | The zip code is specific to each participant and will represent their individual residential location. |
| **Mental Health Priority Issues Variables** | | | |
| Overall Mental Health Importance | The importance attributed to the overall mental health of young people in the community | Ordinal | Categories:   - Not important - Somewhat important - Very important |
| Alcohol and Substance Use Importance | The importance attributed to alcohol, drug, or substance use among young people in the community | Ordinal | Categories:   - Not important - Somewhat important - Very important |
| Depression, Anxiety, and Stress Importance | The importance attributed to depression, anxiety, and stress among young people in the community | Ordinal | Categories:   - Not important - Somewhat important - Very important |
| Schizophrenia & Severe Mental Illness Importance | The importance attributed to schizophrenia and other severe mental illnesses among young people in the community | Ordinal | Categories:   - Not important - Somewhat important - Very important |
| Suicide and Self-Harm Importance | The importance attributed to suicide and self-harm among young people in the community | Ordinal | Categories:   - Not important - Somewhat important - Very important |
| Homelessness Importance | The importance attributed to homelessness among young people in the community | Ordinal | Categories:   - Not important - Somewhat important - Very important |
| Poverty Importance | The importance attributed to poverty among young people in the community | Ordinal | Categories:   - Not important - Somewhat important - Very important |
| Racism and Inequity Importance | The importance attributed to racism and inequity against young people in the community | Ordinal | Categories:   - Not important - Somewhat important - Very important |
| Social Connectedness Importance | The importance attributed to social connectedness among young people in the community | Ordinal | Categories:   - Not important - Somewhat important - Very important |
| **Satisfaction Variables** | | | |
| Satisfaction- Overall Mental Health Efforts | The level of satisfaction with the overall efforts, including those by the community, schools, and the health system, to address the mental health of young people in the community | Ordinal | Categories:   - Not satisfied - Somewhat satisfied - Very satisfied |
| Satisfaction - Alcohol, Drug, or Substance Use Efforts | The level of satisfaction with the overall efforts, including those by the community, schools, and the health system, to address alcohol, drug, or substance use among young people in the community | Ordinal | Categories:   - Not satisfied - Somewhat satisfied - Very satisfied |
| Satisfaction- Depression, Anxiety, and Stress Efforts | The level of satisfaction with the overall efforts, including those by the community, schools, and the health system, to address depression, anxiety, and stress among young people in the community | Ordinal | Categories:   - Not satisfied - Somewhat satisfied - Very satisfied |
| Satisfaction- Schizophrenia and Severe Mental Illnesses Efforts | The level of satisfaction with the overall efforts, including those by the community, schools, and the health system, to address schizophrenia and other severe mental illnesses among young people in the community | Ordinal | Categories:   - Not satisfied - Somewhat satisfied - Very satisfied |
| Satisfaction- Suicide and Self-Harm Efforts | The level of satisfaction with the overall efforts, including those by the community, schools, and the health system, to address suicide and self-harm among young people in the community | Ordinal | Categories:   - Not satisfied - Somewhat satisfied - Very satisfied |
| Satisfaction- Homelessness Efforts | The level of satisfaction with the overall efforts, including those by the community, schools, and the health system, to address homelessness among young people in the community | Ordinal | Categories:   - Not satisfied - Somewhat satisfied - Very satisfied |
| Satisfaction- Poverty Efforts | The level of satisfaction with the overall efforts, including those by the community, schools, and the health system, to address poverty among young people in the community | Ordinal | Categories:   - Not satisfied - Somewhat satisfied - Very satisfied |
| Satisfaction- Racism and Inequity Efforts | The level of satisfaction with the overall efforts, including those by the community, schools, and the health system, to address racism and inequity against young people in the community | Ordinal | Categories:   - Not satisfied - Somewhat satisfied - Very satisfied |
| Satisfaction- Social Connectedness Efforts | The level of satisfaction with the overall efforts, including those by the community, schools, and the health system, to promote social connectedness among young people in the community | Ordinal | Categories:   - Not satisfied - Somewhat satisfied - Very satisfied |
| **Barriers to Mental Health** | | | |
| **Barriers to Mental Health** | The most important barriers to the mental health of young people in the community Participants chose three options. | Nominal | Categories:   - Lack of support from peers - Lack of support from parents/guardians and family - Lack of future economic opportunity (e.g., jobs) - Lack of future educational opportunity (e.g., college) - Lack of after-school activities - Lack of support from the school system - Lack of access to quality health care - Lack of awareness on and skills related to mental health - Exposure to violence - Exposure to racism and social injustice - Unstable or unavailable housing (housing insecurity) - Other (Participants could provide additional barriers not listed above) |
| **Mental Health Intervention Platforms** | | | |
| Ideal Places for Connection | The ideal places to connect with youth and work towards improving youth mental health in Seattle/King County Participants chose two options. | Nominal | Categories:   - Schools - Community centers - Churches - Online - Workplaces - Clinics and other health care settings - In public/on the street - Other (Participants can provide additional places not listed above) |
| Needed Services and Programs | The types of services, programs, or activities most needed to support youth mental health in Seattle/King County Participants chose two options. | Nominal | Categories:   - Positive, youth-led mental health messaging (e.g., messages developed by youth, for youth, about how to stay healthy) - Training in resilience and self-care - Training in awareness and peer support - Provision of safe spaces - Access to counseling and treatment based on my community's values and traditions - Housing and social services - Employment opportunities and career counseling - Other (Participants can provide additional services, programs, or activities not listed above) |
| **Mental Health Intervention Acceptability and Feasibility Variables** | | | |
| Friendship Bench Approval | Approval of use of Friendship Bench to meet the mental health needs of young people in Seattle/King County | Ordinal | Categories:   - Completely disagree - Disagree - Neither agree nor disagree - Agree - Completely agree |
| Friendship Bench Appeal | Appeal of use of Friendship Bench to meet the mental health needs of young people in Seattle/King County | Ordinal | Categories:   - Completely disagree - Disagree - Neither agree nor disagree - Agree - Completely agree |
| Like Friendship Bench | Participants' liking of the Friendship Bench to meet the mental health needs of young people in Seattle/King County | Ordinal | Categories:   - Completely disagree - Disagree - Neither agree nor disagree - Agree - Completely agree |
| Welcome Friendship Bench | Participants' willingness to welcome the implementation of Friendship Bench to meet the mental health needs of young people | Ordinal | Categories:   - Completely disagree - Disagree - Neither agree nor disagree - Agree - Completely agree |
| Gathering spaces Approval | Approval of use of Gathering spaces to meet the mental health needs of young people in Seattle/King County | Ordinal | Categories:   - Completely disagree - Disagree - Neither agree nor disagree - Agree - Completely agree |
| Gathering spaces Appeal | Appeal of the use of Gathering spaces to meet the mental health needs of young people in Seattle/King County | Ordinal | Categories:   - Completely disagree - Disagree - Neither agree nor disagree - Agree - Completely agree |
| Like Gathering spaces | Participants' liking of the implementation of Gathering spaces to meet the mental health needs of young people in Seattle/King County | Ordinal | Categories:   - Completely disagree - Disagree - Neither agree nor disagree - Agree - Completely agree |
| Welcome Gathering spaces | Participants' willingness to welcome the implementation of Gathering spaces to meet the mental health needs of young people | Ordinal | Categories:   - Completely disagree - Disagree - Neither agree nor disagree - Agree - Completely agree |
| Peer support programs Approval | Approval of use of Peer support programs to meet the mental health needs of young people in Seattle/King County | Ordinal | Categories:   - Completely disagree - Disagree - Neither agree nor disagree - Agree - Completely agree |
| Peer support programs Appeal | Appeal of the use of Peer support programs to meet the mental health needs of young people in Seattle/King County | Ordinal | Categories:   - Completely disagree - Disagree - Neither agree nor disagree - Agree - Completely agree |
| Like Peer support programs | Participants' liking of the implementation of Peer support programs to meet the mental health needs of young people in Seattle/King County | Ordinal | Categories:   - Completely disagree - Disagree - Neither agree nor disagree - Agree - Completely agree |
| Welcome Peer support programs | Participants' willingness to welcome the implementation of Peer support programs to meet the mental health needs of young people | Ordinal | Categories:   - Completely disagree - Disagree - Neither agree nor disagree - Agree - Completely agree |
| School-based mental health promotion and prevention activities Approval | Approval of use of School-based mental health promotion and prevention activities to meet the mental health needs of young people in Seattle/King County | Ordinal | Categories:   - Completely disagree - Disagree - Neither agree nor disagree - Agree - Completely agree |
| School-based mental health promotion and prevention activities Appeal | Appeal of use of School-based mental health promotion and prevention activities to meet the mental health needs of young people in Seattle/King County | Ordinal | Categories:   - Completely disagree - Disagree - Neither agree nor disagree - Agree - Completely agree |
| Like School-based mental health promotion and prevention activities | Participants' liking of the implementation of School-based mental health promotion and prevention activities to meet the mental health needs of young people in Seattle/King County | Ordinal | Categories:   - Completely disagree - Disagree - Neither agree nor disagree - Agree - Completely agree |
| Welcome School-based mental health promotion and prevention activities | Participants' willingness to welcome the implementation of School-based mental health promotion and prevention activities to meet the mental health needs of young people | Ordinal | Categories:   - Completely disagree - Disagree - Neither agree nor disagree - Agree - Completely agree |
