# Supplementary material for: A mixed methods analysis of youth mental health intervention feasibility and acceptability in a North American city: Perspectives from Seattle, Washington
Source: PLoS One. 2024 Mar 14;19(3):e0288214. doi: 10.1371/journal.pone.0288214 (PMC10939237; doi:10.1371/journal.pone.0288214)
Supplement: S1 Checklist — (DOCX) [file pone.0288214.s002.docx]

STROBE Statement—checklist of items that should be included in reports of observational studies

|  | Item No. | Recommendation | Page  No. | Relevant text from manuscript |
| --- | --- | --- | --- | --- |
| **Title and abstract** | 1 | (*a*) Indicate the study’s design with a commonly used term in the title or the abstract | Page 1 | *“A mixed methods analysis of youth mental health intervention feasibility and acceptability in a North American city: perspectives from Seattle, Washington”* |
|  |  | (*b*) Provide in the abstract an informative and balanced summary of what was done and what was found | Pages 2-3,  lines 32-59 | *Introduction: In March 2021, the Governor of Washington declared a youth mental health crisis. State data revealed high rates of youth suicide and inadequate access to services. This mixed-methods study examines youth and adult perspectives on mental health service gaps and opportunities in Seattle by assessing needs, feasibility, and acceptability of interventions to support youth mental health.*  *Methods: We interviewed 15 key informants to identify the contextual, structural, and individual-level factors that increase the risk of poor mental health and deter access to care among young people. We complimented these data with a cross-sectional 25-item survey of 117 participants in King County to assess the feasibility and acceptability of interventions for youth mental health. We conducted an inductive thematic qualitative analysis of the interviews and performed descriptive analyses of the quantitative data, using t-tests and χ^2^ tests to summarize and compare participant characteristics stratified by age group.*  *Results: Qualitative informants attributed challenges to youth mental health to social and relational problems. Example interventions included creating environments that increase belonging and implementation of culturally congruent mental health services. Quantitative study participants rated all evidence-based mental health interventions presented as highly acceptable. However, youth preferred interventions promoting social connectedness, peer support, and holistic approaches to care, while non-youth preferred interventions focused on suicide, alcohol, and substance abuse prevention. Both key informants and survey participants identified schools as the highest priority setting for mental health interventions. There were no significant differences among quantitative outcomes.*  *Conclusion: Our findings highlight the need for reducing social isolation and increasing social connectedness to support youth mental health. Schools and digital tools were preferred platforms for implementation. Engaging multiple stakeholders, especially young people, and addressing cultural needs and accessibility of mental health resources are important pre-implementation activities for youth mental health intervention in a US city.* |
| Introduction | | | |  |
| Background/rationale | 2 | Explain the scientific background and rationale for the investigation being reported | Pages 4-5,  lines 60-102 | *Psychosocial stressors induced by the COVID-19 pandemic have precipitated a mental health crisis for young people in cities around the United States. The U.S. Surgeon General issued an advisory on youth mental health in late 2021, signaling the public health significance of the issue and the need for “the nation’s immediate awareness and action (1).” In Seattle, Washington, hospitals and emergency rooms endured unprecedented rates of admission for psychiatric complaints throughout 2021 (2). The state’s Governor declared a youth mental health crisis in March of 2021, marking the need to prioritize youth well-being (2). However, challenges to youth mental health were growing well before the pandemic.*  *In Washington State, the youth suicide rate stood at 11.4 per 100,000 in 2016; however, by 2020, it had risen to 15.7 per 100,000 (3). Currently, approximately 22% of youth aged 15-24 in the state of Washington are estimated to live with a mental disorder, while 12% are estimated to live with a substance abuse disorder (4). However, access to standard mental health services is limited in many parts of the state, given workforce shortages (5).*  *Seattle’s social context, notable for rising rates of gun violence, racial and ethnic disparities in the criminal justice system and in health outcomes, as well as housing instability and homelessness, significantly shapes the mental health trajectories of young people (6-8). Recent policies and programs seek to support the social and mental wellbeing of youth and families. Best Starts for Kids (BSK), an initiative launched in 2015, invests in early child development, and youth and family initiatives throughout King County (9). Seattle is also known for community activism and sustainable urbanization that supports community well-being and works to reduce social risk factors that can contribute to poor mental health outcomes (10).*  *These efforts matter. While urban environments can pose high risks for poor mental health (11-13), they can also offer mental health-promoting environments, institutions, and services that are accessible, affordable, and culturally appropriate to diverse populations, supporting youth mental health through promotion, prevention and care (13-16). Few cities adopt an intersectoral approach to address the mental health needs of adolescents and young adults, taking advantage of resources within and outside of the health system. In response to these needs, the citiesRISE consortium, a global platform committed to transforming the state of mental health policy and practice in cities around the world (17), enables young people to drive action in their communities by positioning them as expert partners with local stakeholders and by creating a unified youth voice. citiesRISE selected Seattle as a primary site to initiate engagement around youth mental health.*  *In 2019, a citiesRISE landscaping assessment of youth mental health identified opportunities to engage and support young people in Seattle. These opportunities included promoting racial equity, expanding the definition of mental health, and addressing substance abuse, workforce development, and peer support among youth. The same year, we hosted a multistakeholder roundtable discussion at the University of Washington to identify priorities and opportunities for supporting youth mental health in Seattle. The current study aims to ascertain in greater detail the kinds of support across the mental health care continuum support recommended by young people and key stakeholders who could assist with implementation. We examine, quantitatively and qualitatively, the feasibility, acceptability of specific mental health interventions for youth in Seattle.* |
| Objectives | 3 | State specific objectives, including any prespecified hypotheses | Page 5,  Lines 98-102 | *The current study aims to ascertain in greater detail the kinds of support across the mental health care continuum support recommended by young people and key stakeholders who could assist with implementation. We examine, quantitatively and qualitatively, the feasibility, acceptability of specific mental health interventions for youth in Seattle.* |
| Methods | | | |  |
| Study design | 4 | Present key elements of study design early in the paper | Page 5-6,  Lines 105-115 | *We used a mixed methods study design to survey public policy informants, young people, and adults in Seattle/King County a) on the contextual, structural, and individual level factors that place young people at risk for poor mental health and deter access to care, b) to identify opportunities for interventions, and c) to determine feasibility and acceptability of interventions that could support youth mental health.*  *We employed these methods:*   1. *Qualitative key-informant interviews with 15 research participants representing youth and public policymakers and* 2. *A cross-sectional, quantitative anonymous survey, distributed via Instagram, of 94 young people and 12 adults in the Seattle region with the option for open-ended feedback.* |
| Setting | 5 | Describe the setting, locations, and relevant dates, including periods of recruitment, exposure, follow-up, and data collection | Page 6,  line 123-127  Page 7, lines 149-150  Page 7  lines 150-151 | *We interviewed participants with knowledge of mental health employed in public service (city and county government) and young people (17 years and older) living in King County by recruiting a convenience sample of 15 key informants between September and December 2019. We recruited five adult key-informants in public service during a series of meetings to orient local policy makers and mental health experts to citiesRISE activities and aims.*  *We invited young people and adults in the Seattle/King County area to complete brief, structured, online questionnaires from February through March 2020.*  *Questionnaires took approximately fifteen minutes to be complete and were available in English. Data collection occurred between September 2019 and March 2020.* |
| Participants | 6 | (*a*) *Cohort study*—Give the eligibility criteria, and the sources and methods of selection of participants. Describe methods of follow-up  *Case-control study*—Give the eligibility criteria, and the sources and methods of case ascertainment and control selection. Give the rationale for the choice of cases and controls  *Cross-sectional study*—Give the eligibility criteria, and the sources and methods of selection of participants | Page 6,  lines 123-130  Page 7,  lines 148-155 | *We interviewed participants with knowledge of mental health employed in public service (city and county government) and young people (17 years and older) living in King County by recruiting a convenience sample of 15 key informants between September and December 2019. We recruited five adult key-informants in public service during a series of meetings to orient local policy makers and mental health experts to citiesRISE activities and aims. We used snow-ball sampling through youth networks known to the research team members to recruit youth key-informants from two different groups: 1) young people engaged in youth-focused initiatives in Seattle (N= 4) and 2) young people working or studying in the Seattle community (N=6).*  *We invited young people and adults in the Seattle/King County area to complete brief, structured, online questionnaires from February through March 2020. Participants were recruited through fliers and links that were disseminated through school- and community organization-based mailing lists and through an Instagram promotion targeting young people in the Seattle/King County region aged 17-24. Adults over the age of 24 were also eligible to participate.* |
|  |  | (*b*) *Cohort study*—For matched studies, give matching criteria and number of exposed and unexposed  *Case-control study*—For matched studies, give matching criteria and the number of controls per case |  | *Not applicable* |
| Variables | 7 | Clearly define all outcomes, exposures, predictors, potential confounders, and effect modifiers. Give diagnostic criteria, if applicable | Pages 8-10,  lines 162-213  S1 Appendix | *Measures*  *Demographic information included participant age (in years), gender identity (woman, man, nonbinary), race or ethnicity (Alaska Native, American Indian/Native American, Asian, Asian – South Asian, Asian – Southeast Asian, Black/African American, Black African, Hispanic/Latinx/Chicanx, Middle Eastern, Native Hawaiian, Pacific Islander, White/European American, as well as any racial/ethnic identity not listed), youth identity status (youth or non-youth), birth year, employment status (studying, employed, self-employed/freelance, interning, part-time, unemployed-looking for work, unemployed-not looking for work, homemakers, military, retired, not able to work, or other), education level (no formal education, pre-school to 8th grade, some high school with no diploma, high school graduate with a diploma or equivalent (e.g., GED), some college credit with no degree, trade/technical/vocational training, associate degree, Bachelor's degree, Master's degree, Professional degree, Doctorate degree), South Seattle residence (yes or no), and zip code.*  *Mental Health Priority Issues and Satisfaction. Relative priority of nine different mental health-related issues (overall mental health, alcohol and substance use, depression, anxiety and stress, schizophrenia and severe mental illness, suicide and self-harm, homelessness, poverty, racism and inequality, and social connectedness) was assessed with a three-level Likert scale, where each issue was rated as not important, somewhat important, or very important. Satisfaction with these same nine mental health-related issues was assessed with another three-level Likert scale, where respondents rated their perception of efforts to address each issue as not satisfied, somewhat satisfied, or very satisfied.*  *Barriers to Mental Health. Participants were presented with a list of potential barriers to youth mental health (lack of support from peers, lack of support from parents/guardians and family, lack of future economic opportunity (e.g., jobs), lack of future educational opportunity (e.g., college), lack of after-school activities, lack of support from the school system, lack of access to quality health care, lack of awareness and skills related to mental health, exposure to violence, exposure to racism and social injustice, unstable or unavailable housing (housing insecurity), and other barriers not listed above as identified by participants.) and asked to choose the three most important barriers.*  *Mental Health Intervention Platforms. Preferences for mental health intervention platforms (schools, community centers, churches, online platforms, workplaces, clinics and other health care settings, in public/on the street, or other places not listed above as identified by participants) and approaches to improve youth mental health (positive, youth-led mental health messaging developed by youth for youth, training in resilience and self-care, training in awareness and peer support, provision of safe spaces, access to counseling and treatment aligned with the values and traditions of the community, housing and social services, employment opportunities and career counseling, or other services, programs, or activities not listed above as identified by participants) were assessed by asking respondents to choose two ideal platforms and two priority services.*  *Acceptability of Intervention Measure (AIM). Four specific evidence-based interventions or services were described in detail, and acceptability of each intervention or service was assessed using the 4-item Acceptability of Intervention Measure (AIM) (18).These interventions included the Friendship Bench psychosocial support model, the provision of safe spaces, peer navigation and support services, and arts-based mental health promotion and suicide prevention in schools. The items specifically asked participants to indicate the level of approval (e.g., "Friendship Bench would meet my approval"), appeal (e.g., "Friendship Bench would be appealing to me"), likeness (e.g., "I would like Friendship Bench"), and willingness to welcome (e.g., "I would welcome Friendship Bench") the interventions. Participants rated their level of agreement or disagreement with each statement using a 5-point Likert scale, ranging from "completely disagree" to " completely agree." The scale provides a numerical rating that reflects the participant's perception of the intervention's acceptability.* |
| Data sources/ measurement | 8* | For each variable of interest, give sources of data and details of methods of assessment (measurement). Describe comparability of assessment methods if there is more than one group | Pages 8-10,  lines 162-213  S1 Appendix | *Measures*  *Demographic information included participant age (in years), gender identity (woman, man, nonbinary), race or ethnicity (Alaska Native, American Indian/Native American, Asian, Asian – South Asian, Asian – Southeast Asian, Black/African American, Black African, Hispanic/Latinx/Chicanx, Middle Eastern, Native Hawaiian, Pacific Islander, White/European American, as well as any racial/ethnic identity not listed), youth identity status (youth or non-youth), birth year, employment status (studying, employed, self-employed/freelance, interning, part-time, unemployed-looking for work, unemployed-not looking for work, homemakers, military, retired, not able to work, or other), education level (no formal education, pre-school to 8th grade, some high school with no diploma, high school graduate with a diploma or equivalent (e.g., GED), some college credit with no degree, trade/technical/vocational training, associate degree, Bachelor's degree, Master's degree, Professional degree, Doctorate degree), South Seattle residence (yes or no), and zip code.*  *Mental Health Priority Issues and Satisfaction. Relative priority of nine different mental health-related issues (overall mental health, alcohol and substance use, depression, anxiety and stress, schizophrenia and severe mental illness, suicide and self-harm, homelessness, poverty, racism and inequality, and social connectedness) was assessed with a three-level Likert scale, where each issue was rated as not important, somewhat important, or very important. Satisfaction with these same nine mental health-related issues was assessed with another three-level Likert scale, where respondents rated their perception of efforts to address each issue as not satisfied, somewhat satisfied, or very satisfied.*  *Barriers to Mental Health. Participants were presented with a list of potential barriers to youth mental health (lack of support from peers, lack of support from parents/guardians and family, lack of future economic opportunity (e.g., jobs), lack of future educational opportunity (e.g., college), lack of after-school activities, lack of support from the school system, lack of access to quality health care, lack of awareness and skills related to mental health, exposure to violence, exposure to racism and social injustice, unstable or unavailable housing (housing insecurity), and other barriers not listed above as identified by participants.) and asked to choose the three most important barriers.*  *Mental Health Intervention Platforms. Preferences for mental health intervention platforms (schools, community centers, churches, online platforms, workplaces, clinics and other health care settings, in public/on the street, or other places not listed above as identified by participants) and approaches to improve youth mental health (positive, youth-led mental health messaging developed by youth for youth, training in resilience and self-care, training in awareness and peer support, provision of safe spaces, access to counseling and treatment aligned with the values and traditions of the community, housing and social services, employment opportunities and career counseling, or other services, programs, or activities not listed above as identified by participants) were assessed by asking respondents to choose two ideal platforms and two priority services.*  *Acceptability of Intervention Measure (AIM). Four specific evidence-based interventions or services were described in detail, and acceptability of each intervention or service was assessed using the 4-item Acceptability of Intervention Measure (AIM) (18).These interventions included the Friendship Bench psychosocial support model, the provision of safe spaces, peer navigation and support services, and arts-based mental health promotion and suicide prevention in schools. The items specifically asked participants to indicate the level of approval (e.g., "Friendship Bench would meet my approval"), appeal (e.g., "Friendship Bench would be appealing to me"), likeness (e.g., "I would like Friendship Bench"), and willingness to welcome (e.g., "I would welcome Friendship Bench") the interventions. Participants rated their level of agreement or disagreement with each statement using a 5-point Likert scale, ranging from "completely disagree" to " completely agree." The scale provides a numerical rating that reflects the participant's perception of the intervention's acceptability.* |
| Bias | 9 | Describe any efforts to address potential sources of bias | Pages 30-31,  lines 616-628 | *There are several limitations to our study. First, our quantitative sample size was relatively small, with a total of 117 participants. As such, our findings may not be representative of broader views on youth mental health intervention feasibility and acceptability across Seattle. Participants in our quantitative study were recruited through mental health networks and digital platforms and were likely to have a particular interest in mental health, raising concerns of selection bias. Second, our quantitative assessment centered on a limited number of evidence-based interventions (EBIs) and did not include social and structural interventions that youth also valued. Despite the limitations, our quantitative sample captured the racial and ethnic diversity of Seattle, possibly enhancing the diversity of perspectives. Our mixed method approach and triangulation of data and contextual information (e.g., roundtable discussion) served as the primary efforts to address bias. The multistakeholder participants in this study displayed considerable agreement across findings that reinforced intervention directions for youth at greater risk of mental health disparities in an urban setting.* |
| Study size | 10 | Explain how the study size was arrived at | Page 6,  lines 123-130  Page 7,  lines 149-155 | *We interviewed participants with knowledge of mental health employed in public service (city and county government) and young people (17 years and older) living in King County by recruiting a convenience sample of 15 key informants between September and December 2019. We recruited five adult key-informants in public service during a series of meetings to orient local policy makers and mental health experts to citiesRISE activities and aims. We used snow-ball sampling through youth networks known to the research team members to recruit youth key-informants from two different groups: 1) young people engaged in youth-focused initiatives in Seattle (N= 4) and 2) young people working or studying in the Seattle community (N=6). (We estimated that we would achieve saturation with this number of participants.)*  *We invited young people and adults in the Seattle/King County area to complete brief, structured, online questionnaires from February through March 2020. Participants were recruited through fliers and links that were disseminated through school- and community organization-based mailing lists and through an Instagram promotion targeting young people in the Seattle/King County region aged 17-24. Adults over the age of 24 were also eligible to participate.* |

Continued on next page

| Quantitative variables | 11 | Explain how quantitative variables were handled in the analyses. If applicable, describe which groupings were chosen and why | Page 10,  lines 215-218 | *Descriptive analyses, including t tests and χ2 tests, were conducted to summarize and compare participant characteristics stratified by age group (i.e., self-reported youth vs non-youth). Proportions were calculated for categorical responses. AIM summary scores were created by averaging respective item responses and compared across age strata using t tests. All analyses were performed in Stata and RStudio 2022.07.2 (19, 20).* |
| --- | --- | --- | --- | --- |
| Statistical methods | 12 | (*a*) Describe all statistical methods, including those used to control for confounding | Page 10,  lines 215-218 | *Descriptive analyses, including t tests and χ2 tests, were conducted to summarize and compare participant characteristics stratified by age group (i.e., self-reported youth vs non-youth). Proportions were calculated for categorical responses. AIM summary scores were created by averaging respective item responses and compared across age strata using t tests. All analyses were performed in Stata and RStudio 2022.07.2 (19, 20).* |
|  |  | (*b*) Describe any methods used to examine subgroups and interactions | Page 10,  lines 215-218 | *Descriptive analyses, including t tests and χ2 tests, were conducted to summarize and compare participant characteristics stratified by age group (i.e., self-reported youth vs non-youth). Proportions were calculated for categorical responses. AIM summary scores were created by averaging respective item responses and compared across age strata using t tests. All analyses were performed in Stata and RStudio 2022.07.2 (19, 20).* |
|  |  | (*c*) Explain how missing data were addressed | Table 2  Page 21, lines 444-448 | S2 Table: Questionnaire participant characteristics stratified by youth vs. non-youth (n=117)   \|  \| Missing \| Overall \|  \| Non-Youth \| Youth \| p \| \| --- \| --- \| --- \| --- \| --- \| --- \| --- \| \| **n** \|  \| 117 \|  \| 12 \| 94 \|  \| \| **Woman (%)** \| 0 \| 87 (74.4) \|  \| 9 (75.0) \| 78 (83.0) \| 0.78 \| \| **Non-Binary (%)** \| 0 \| 9 (7.7) \|  \| 1 (8.3) \| 8 (8.5) \| 1 \| \| **Age** \| 65 \|  \|  \|  \|  \|  \| \| 17-19 (%) \|  \| 14 (26.9) \|  \| 0 (0.0) \| 13 (31.0) \|  \| \| 20-24 (%) \|  \| 29 (55.8) \|  \| 5 (55.6) \| 24 (57.1) \|  \| \| 25-34 (%) \|  \| 6 (11.5) \|  \| 1 (11.1) \| 5 (11.9) \|  \| \| 45-54 (%) \|  \| 1 (1.9) \|  \| 1 (11.1) \| 0 (0.0) \|  \| \| 55-64 (%) \|  \| 2 (3.8) \|  \| 2 (22.2) \| 0 (0.0) \|  \| \| **Race/Ethnicity** \|  \|  \|  \|  \|  \|  \| \| AI/AN (%) \| 0 \| 1 (0.9) \|  \| 0 (0.0) \| 1 (1.1) \| 1 \| \| Asian (%) \| 0 \| 18 (15.4) \|  \| 1 (8.3) \| 16 (17.0) \| 0.723 \| \| South Asian (%) \| 0 \| 5 (4.3) \|  \| 1 (8.3) \| 4 (4.3) \| 1 \| \| South-East Asian (%) \| 0 \| 11 (9.4) \|  \| 0 (0.0) \| 11 (11.7) \| 0.454 \| \| Black American (%) \| 0 \| 7 (6.0) \|  \| 2 (16.7) \| 5 (5.3) \| 0.382 \| \| Black African (%) \| 0 \| 2 (1.7) \|  \| 1 (8.3) \| 1 (1.1) \| 0.538 \| \| Latinx (%) \| 0 \| 3 (2.6) \|  \| 1 (8.3) \| 2 (2.1) \| 0.767 \| \| Middle Eastern (%) \| 0 \| 5 (4.3) \|  \| 0 (0.0) \| 5 (5.3) \| 0.924 \| \| Hawaiian (%) \| 0 \| 2 (1.7) \|  \| 0 (0.0) \| 2 (2.1) \| 1 \| \| Pacific Islander (%) \| 0 \| 5 (4.3) \|  \| 1 (8.3) \| 4 (4.3) \| 1 \| \| White (%) \| 0 \| 57 (48.7) \|  \| 5 (41.7) \| 52 (55.3) \| 0.558 \| \| **Employed (%)** \| 0 \| 50 (42.7) \|  \| 8 (66.7) \| 42 (44.7) \| 0.259 \| \| **Studying (%)** \| 0 \| 65 (55.6) \|  \| 5 (41.7) \| 59 (62.8) \| 0.274 \| \| **Living in South Seattle (%)** \| 0 \| 21 (17.9) \|  \| 6 (50.0) \| 15 (16.0) \| 0.016 \|   *The table displays the demographic characteristics of the questionnaire participants, including their sex, age, race/ethnicity, employment status, education status, and residence status (i.e., living in south Seattle). Age data were missing for 65 participants who completed a REDCap version of the survey. T-tests and χ^2^ tests were used to compare participant characteristics by age designation.* |
|  |  | (*d*) *Cohort study*—If applicable, explain how loss to follow-up was addressed  *Case-control study*—If applicable, explain how matching of cases and controls was addressed  *Cross-sectional study*—If applicable, describe analytical methods taking account of sampling strategy | Page 10,  lines 215-218 | *Descriptive analyses, including t tests and χ2 tests, were conducted to summarize and compare participant characteristics stratified by age group (i.e., self-reported youth vs non-youth). Proportions were calculated for categorical responses. AIM summary scores were created by averaging respective item responses and compared across age strata using t tests. All analyses were performed in Stata and RStudio 2022.07.2 (19, 20).* |
|  |  | (*e*) Describe any sensitivity analyses |  | Not applicable |
| Results | | | | |
| Participants | 13* | (a) Report numbers of individuals at each stage of study—e.g. numbers potentially eligible, examined for eligibility, confirmed eligible, included in the study, completing follow-up, and analysed | Page 13,  lines 252-256  Pages  20-21,  lines 437-442  Table 2  Page 21, lines 444-448 | *We conducted qualitative interviews with 15 key informants: 5 public service informants and 10 young people or youth allies. Among the public service informants, all served in city or county government roles. Youth participants ranged in age from 17 - 31, with 7 participants under the age of 25. The group included 3 men and 7 women and represented young people with multiple cultural identities.*  *One-hundred and seventeen participants completed the quantitative survey of intervention acceptability and feasibility (****Table 2****). Ninety-four (80%) identified as youth (e.g., under 35 years). Most (87, 74%) were women. Participants were broadly representative of the Seattle/King County population in terms of race: 57 (49%) were white, 18 (15%) were Asian, 11 (9%) were Southeast Asian, and 7 (6%) were Black American. Fifty (43%) were employed, and 65 (56%) were currently studying.* |
|  |  | (b) Give reasons for non-participation at each stage |  | Not applicable |
|  |  | (c) Consider use of a flow diagram |  | Not applicable |
| Descriptive data | 14* | (a) Give characteristics of study participants (eg demographic, clinical, social) and information on exposures and potential confounders | Page 13,  lines 252-256  Pages  20-21,  lines 437-442  Table 2  Page 21, lines 444-448 | *We conducted qualitative interviews with 15 key informants - 5 public service informants and 10 young people or youth allies. Among the public service informants, all served in city or county government roles. Youth participants ranged in age from 17 - 31, with 7 participants under the age of 25. The group included 3 men and 7 women and represented young people with multiple cultural identities.*  *One-hundred and seventeen participants completed the quantitative survey of intervention acceptability and feasibility (****Table 2****). Ninety-four (80%) identified as youth (e.g., under 35 years). Most (87, 74%) were women. Participants were broadly representative of the Seattle/King County population in terms of race: 57 (49%) were white, 18 (15%) were Asian, 11 (9%) were Southeast Asian, and 7 (6%) were Black American. Fifty (43%) were employed, and 65 (56%) were currently studying.* |
|  |  | (b) Indicate number of participants with missing data for each variable of interest | Pages  7-8,  lines 159-161  Page 10,  lines 219-221  Table 2  Page 21, lines 444-448  Table 3  Page 22, lines 482-485 | *An error in the REDCap survey meant that data on age in years are missing for n=65 participants, though all participants self-reported being over the age of 16, and all self-identified as “youth” or “non-youth.”*  *In addition to the age-related data missingness identified above, there was a moderate amount of missingness due to incomplete survey responses by participants. Given that only descriptive statistical approaches were used, complete case analysis was deemed appropriate.*  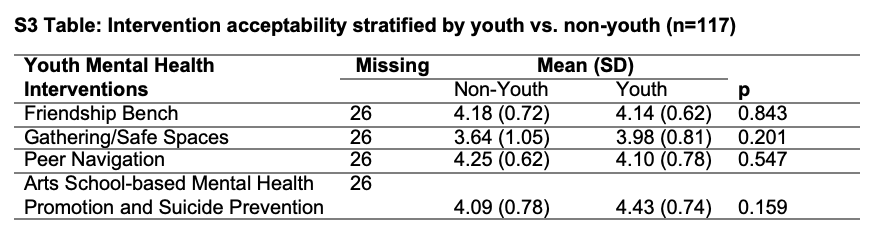  *Means, standard deviations (SDs), and t-test comparison p-values are reported for participant acceptability of intervention measure (AIM) scores for each mental health intervention across self-reported youth strata (range: 1-5).* |
|  |  | (c) *Cohort study*—Summarise follow-up time (eg, average and total amount) |  | Not applicable |
| Outcome data | 15* | *Cohort study*—Report numbers of outcome events or summary measures over time |  | Not applicable |
|  |  | *Case-control study—*Report numbers in each exposure category, or summary measures of exposure |  | Not applicable |
|  |  | *Cross-sectional study—*Report numbers of outcome events or summary measures | S1 Appendix  Table 2  Page 21, lines 444-448  Table 3  Page 22, lines 482-485 |  |
| Main results | 16 | (*a*) Give unadjusted estimates and, if applicable, confounder-adjusted estimates and their precision (eg, 95% confidence interval). Make clear which confounders were adjusted for and why they were included | Pages  10-25  lines 224-498 | Refer to results section of manuscript |
|  |  | (*b*) Report category boundaries when continuous variables were categorized |  | Not applicable |
|  |  | (*c*) If relevant, consider translating estimates of relative risk into absolute risk for a meaningful time period |  | Not applicable |

Continued on next page

| Other analyses | 17 | Report other analyses done—eg analyses of subgroups and interactions, and sensitivity analyses |  | *Not applicable* | |
| --- | --- | --- | --- | --- | --- |
| Discussion | | | | |  |
| Key results | 18 | Summarise key results with reference to study objectives | Page 26,  Lines 500-512 | *This study identified gaps in youth mental health services and opportunities for intervention; and assessed the feasibility and acceptability of interventions that could support youth mental health in Seattle. Our qualitative key informants--young people/youth allies and public servants--emphasized unmet needs to reduce social isolation and lack of connectedness as key contributors to poor mental health among youth in Seattle. Quantitative survey data confirmed the need for greater access to mental health services and revealed differences in perceived acceptability of specific interventions between youth and non-youth participants. While both groups viewed evidence-based mental health interventions as highly acceptable, youth preferred interventions promoting social connectedness, peer support, and holistic approaches to care, while non-youth were more concerned with interventions focused on suicide, alcohol and substance abuse prevention. Youth participants also emphasized the significance of their active involvement in the development and implementation of mental health interventions, as it would improve relevance of the interventions to their needs.* | |
| Limitations | 19 | Discuss limitations of the study, taking into account sources of potential bias or imprecision. Discuss both direction and magnitude of any potential bias | Pages 30-31,  lines 615-628 | *There are several limitations to our study. First, our quantitative sample size was relatively small, with a total of 117 participants. As such, our findings may not be representative of broader views on youth mental health intervention feasibility and acceptability across Seattle. Participants in our quantitative study were recruited through Instagram, raising concerns of selection bias. Second, our quantitative assessment centered on a limited number of evidence-based interventions (EBIs) and did not include social and structural interventions that youth also valued. Despite these limitations, the multistakeholder participants in this study provided useful triangulation of findings that reinforced intervention directions for youth at greater risk of mental health disparities in an urban setting.* | |
| Interpretation | 20 | Give a cautious overall interpretation of results considering objectives, limitations, multiplicity of analyses, results from similar studies, and other relevant evidence | Page 31,  lines 630-643 | *This study highlights the need for interventions that support youth mental health in Seattle, particularly those that reduce social isolation and increase social connectedness. These problems have intensified during the years of the pandemic, and cities play a role in meeting these mental health needs. Schools and digital platforms were identified as preferred platforms for interventions, while psychological interventions and peer-to-peer support were the preferred mental health interventions. Engaging multiple sectors in the implementation of mental health interventions (e.g., education and health) and social interventions that can support mental health is a starting point.*  *Equally important, involving young people in the design and implementation of these interventions can improve their acceptability and uptake. Policymakers and mental health service providers should prioritize mental health literacy training and awareness-raising opportunities to support meaningful youth participation in mental health initiatives. Embracing youth-designed mental health interventions fosters shared participation and empowers young people to drive solutions that address their mental health needs.* | |
| Generalisability | 21 | Discuss the generalisability (external validity) of the study results | Page 26,  lines 512-517 | *Given the regionally specific context of our study, generalizability to other populations may be limited. Nonetheless, our findings shed light on the concerns voiced by young individuals in Seattle, which align with those expressed by young people in other studies involving peer support, school-based interventions, and digital mental health interventions as well as the need for safe spaces for building connections in urban environments.* | |
| Other information | |  | | |  |
| Funding | 22 | Give the source of funding and the role of the funders for the present study and, if applicable, for the original study on which the present article is based |  | *This work was supported in part by funding from Pivotal Ventures and Rural India Supporting Trust to citiesRISE (MS and MH). PYC, TC, ISC, and CGK were supported in part by funding from Pivotal Ventures through a sub-contract with citiesRISE. The*  *funders had no role in the study design, data collection and analysis, decision to publish, or preparation of the manuscript.* | |

*Give information separately for cases and controls in case-control studies and, if applicable, for exposed and unexposed groups in cohort and cross-sectional studies.

**Note:** An Explanation and Elaboration article discusses each checklist item and gives methodological background and published examples of transparent reporting. The STROBE checklist is best used in conjunction with this article (freely available on the Web sites of PLoS Medicine at http://www.plosmedicine.org/, Annals of Internal Medicine at http://www.annals.org/, and Epidemiology at http://www.epidem.com/). Information on the STROBE Initiative is available at www.strobe-statement.org.
